# Extracellular Vesicles from Regenerating Skeletal Muscle Mitigate Muscle Atrophy in an Amyotrophic Lateral Sclerosis Mouse Model

**DOI:** 10.3390/cells14060464

**Published:** 2025-03-20

**Authors:** Jinghui Gao, Aria Sikal, Rachel Hankin, Yaochao Zheng, Elijah Sterling, Kenny Chan, Yao Yao

**Affiliations:** 1 Regenerative Bioscience Center, Department of Animal and Dairy Science, College of Agricultural and Environmental Science, University of Georgia, Athens, GA 30602, USA; 2 Department of Physiology and Pharmacology, College of Veterinary Medicine, University of Georgia, Athens, GA 30602, USA

**Keywords:** amyotrophic lateral sclerosis, regenerating skeletal muscle, extracellular vesicles, muscle atrophy

## Abstract

Amyotrophic lateral sclerosis (ALS) is a devastating neuromuscular disease characterized by progressive motor neuron degeneration and muscle atrophy, with no effective treatments available. Chronic inflammation, which impairs muscle regeneration and promotes proteolysis, is a key contributor to ALS-related muscle atrophy and a promising therapeutic target. Here, we applied extracellular vesicles (EVs) derived from regenerating skeletal muscles 14 days post-acute injury (CTXD14SkM-EVs), which possess a unique anti-inflammatory profile, to target muscle defects in ALS. We found that CTXD14SkM-EVs enhanced myoblast differentiation and fusion in a cellular muscle-wasting model induced by pro-inflammatory cytokine tumor necrosis factor alpha. Intramuscular administration of these EVs into an ALS mouse model mitigated muscle atrophy by promoting muscle regeneration, shifting macrophage polarization from pro-inflammatory M1 to anti-inflammatory M2 state, and suppressing the aberrant Nuclear Factor Kappa B (NF-κB) signaling, a key driver of muscle protein degradation. These results underscore the therapeutic potential of regenerating muscle-derived EVs for combating muscle atrophy in ALS.

## 1. Introduction

Amyotrophic lateral sclerosis (ALS) is a debilitating neuromuscular disease characterized by the progressive degeneration of motor neurons in the spinal cord and brain, accompanied by severe skeletal muscle atrophy [1]. ALS patients experience rapid muscle wasting, and the majority succumb to the disease within 2–5 years of diagnosis, primarily due to respiratory failure. The annual incidence of ALS ranges from 1 to 2.6 cases per 100,000 persons, with a prevalence of approximately 11.8 per 100,000 in the United States [2,3]. Despite extensive research, effective therapeutic options for ALS remain unavailable. Emerging studies indicate that skeletal muscle defects in ALS, beyond being a consequence of motor neuron loss, can retrogradely impair motor neuron health. This bidirectional role contributes to disease progression, positioning skeletal muscle as an accessible and promising therapeutic target [4].

Dysregulated inflammation in ALS-affected skeletal muscle is a significant pathological factor that disrupts the local cellular microenvironment and destabilizes the balance between protein synthesis and degradation [5,6]. Aberrant activation of inflammatory signaling pathways, including the Nuclear Factor Kappa B (NF-κB) pathway, drives this imbalance by promoting muscle protein breakdown and accelerating muscle wasting [7,8]. Furthermore, chronic inflammation hampers muscle regeneration by impairing the regenerative capacity of muscle stem cells and promoting fibrosis and fat infiltration [9]. Notably, sustained inflammation prevents the transition of macrophages from a pro-inflammatory (M1 macrophage) to an anti-inflammatory (M2 macrophage) phenotype [9]. Instead, macrophages adopt a hybrid profile that is unable to effectively clear damaged tissue debris, inhibit angiogenesis, and suppress muscle stem cell activity necessary for tissue repair. Moreover, these macrophages may release profibrotic factors, such as transforming growth factor-beta (TGF-β), which exacerbate pathological fibrosis [10]. Intramuscular administration of the anti-inflammatory cytokine IL-10 has been shown to reduce muscle atrophy in ALS mice by facilitating macrophage polarization and activating muscle stem cells [11]. In this study, we investigate a therapeutic strategy targeting the inflammatory microenvironment in ALS-afflicted skeletal muscle, with the goal of mitigating muscle degradation and promoting regeneration as a potential treatment for ALS.

Skeletal muscle possesses an intrinsic ability to regenerate in response to acute injuries. This process is well-coordinated, involving several interrelated and time-sensitive phases, such as necrosis of injured muscle cells, inflammation, regeneration, maturation, and, ultimately, functional recovery. A single intramuscular injection of the snake venom toxin cardiotoxin (CTX) is widely used to induce acute skeletal muscle regeneration in mice, and a distinctive anti-inflammatory environment emerges by day 14 post-injection (CTXD14SkM) [12]. At this stage, coordinated interactions among various cell types actively support muscle repair. Predominant among these are anti-inflammatory M2 macrophages, which secrete cytokines such as IL-10 and growth factors that drive muscle stem cell differentiation and the maturation of new myofibers [13,14]. Furthermore, M2 macrophages aid in resolving inflammation by suppressing the pro-inflammatory signaling that predominates in the initial phases of injury [15].

EVs, essential mediators of intercellular communication, inherit bioactive molecules such as proteins and nucleic acids from their parent cells. This enables them to mirror the functional characteristics of their cells of origin, facilitating diverse biological effects [16]. EVs secreted by cells within the anti-inflammatory milieu of CTXD14SkM, including M2 macrophages and other immune cells, myogenic progenitor cells, and regenerating myofibers, are likely enriched with factors possessing anti-inflammatory properties. Therefore, in this study, we isolate CTXD14SkM-EVs and assess their therapeutic potential in mitigating inflammation and promoting muscle regeneration and repair in an ALS mouse model.

Here, we showed that CTXD14SkM-EVs enhance myoblast differentiation and fusion in a muscle atrophy cellular model induced by the pro-inflammatory cytokine tumor necrosis factor alpha (TNF-α), highlighting their anti-inflammatory and myogenesis-promoting potential. Furthermore, intramuscular administration of CTXD14SkM-EVs effectively mitigated muscle atrophy and increased muscle fiber size in a well-established ALS mouse model with denervated muscle atrophy. Notably, EV-treated mice exhibited an increased number of regenerating myofibers, accompanied by elevated expression of key myogenic regulatory factors, indicating active muscle regeneration. Additionally, EV treatment facilitated a shift in macrophage polarization from the pro-inflammatory M1 state to the anti-inflammatory M2 state and suppressed activation of the pro-inflammatory NF-κB signaling pathway observed in ALS-afflicted skeletal muscles. These findings underscore the therapeutic potential of EVs derived from regenerating muscle in alleviating inflammation and enhancing muscle regeneration, offering a promising strategy for treating muscle atrophy associated with ALS.

## 2. Materials and Methods

### 2.1. Animals and Treatment

Experiments were conducted using transgenic mice overexpressing human SOD1 with a Gly93-Ala mutation (SOD1^G93A^) (strain designation B6SJL–TgN[SOD1–G93A]1Gur, stock number 002726) and wild-type (WT) B6SJL mice, both obtained from Jackson Laboratories (Bar Harbor, ME, USA). All animals were maintained in a controlled environment under standard laboratory conditions. Five SOD1^G93A^ mice received an intramuscular injection of about 4.4 × 10^9^ CTXD14SkM-EVs suspended in 20 µL phosphate-buffered saline (PBS) (cytiva, Malborough, MA, USA, SH30028.03) into the tibialis anterior (TA) and gastrocnemius (GAS) muscles of one limb. As a control, 20 µL of PBS was injected into the TA and GAS muscles of the opposite limb. Treatments were administered weekly, beginning at the pre-symptomatic stage (day 66), with muscle tissue collected at the late symptomatic stage (day 119).

### 2.2. CTXD14SkM-EVs Isolation

To isolate CTXD14SkM-EVs from acutely injured skeletal muscle, cardiotoxin (CTX) (10 µM, 20 µL) (Sigma-Aldrich, St. Louis, MO, USA, 217503) was injected intramuscularly into the TA and GAS muscles of approximately 2-month-old WT mice. Fourteen days post-injury, the TA and GAS muscles were harvested and sectioned into 2–3 mm pieces using a scalpel. The sliced muscles were then placed in a digestion solution containing 2 mg/mL Collagenase Type II (Worthington-Biochem, Lackwood, NJ, USA, LS004176) in DMEM with 100 U/mL Penicillin/Streptomycin (P-S) (Gibco, New York, NY, USA, 15140122), with 500 µL used for each TA muscle and 1 mL for each GAS muscle. The tissue was rotated in the digestion solution at 37 °C for 24 h. After digestion, the tissue mixture was centrifuged at 1000× *g* for 30 min at 4 °C, and the supernatant was collected and filtered through a 0.8 µm filter (Sigma-Aldrich, St. Louis, MO, USA, SLAAR33SS). CTXD14SkM-EVs were then isolated from the filtered supernatant by ultracentrifugation using ultracentrifugation tubes (Thermo Fisher Scientific, Waltham, MA, USA, 45-239) at 100,000× *g* for 1 h at 4 °C. The resulting EV pellet was resuspended in PBS and stored at −80 °C until use.

### 2.3. MemGlow Assessment

The size and concentration of the isolated EVs were assessed using nanoflow cytometry on the Flow NanoAnalyzer (NanoFCM, Xiamen, China). To determine the proportion of particles with a lipid membrane, the EVs (around 2.4 × 10^8^ EVs diluted in 1 mL PBS) were stained with 2 nM MemGlow 488 (Cytoskeleton, Denver, CO, USA, MG01-02), a fluorogenic membrane probe, for 10 min. Fluorescence was then measured using the Flow NanoAnalyzer. The EV size and concentration were measured at the same dilution using the Flow NanoAnalyzer to ensure consistency. Two mice (both TA and GAS muscles) were used to isolate CTXD14SkM-EVs, with a total yield of approximately 5 × 10^11^ EVs per mouse.

### 2.4. Transmission Electron Microscope (TEM) Imaging Assay

EV suspensions were placed onto glow-discharged, 400-mesh carbon-only copper grids and incubated for 15 min to allow adsorption. Excess liquid was carefully removed using filter paper, followed by negative staining with 3% phosphotungstic acid (PTA, pH 7.0) for 15 s. The stain was gently blotted, and the grids were air-dried at room temperature. Imaging was performed using a JEM-1011 transmission electron microscope (JEOL, Tokyo, Japan) operating at 100 kV. Representative images were acquired, and EV size and morphology were analyzed using ImageJ software version 1.53K.

### 2.5. C2C12 Differentiation

C2C12 myoblast (ATCC, Manassas, VA, USA, CRL-1772™) were cultured in Dulbecco’s Modified Eagle Medium (DMEM; Thermo Fisher Scientific, Waltham, MA, USA) supplemented with 10% FBS and 100 U/mL P-S. Cells were maintained at 37 °C in a humidified incubator with a 5% CO_2_/95% air atmosphere. C2C12 cells were seeded at 100,000 cells per well in the 24-well plate with growth medium. After 24 h, the growth medium was replaced with a differentiation medium (DMEM supplemented with 2% horse serum and 100 U/mL P-S) to initiate differentiation. Three treatment conditions were applied: (1) differentiation medium only, (2) differentiation medium with 5 ng/mL TNF-α and PBS, (3) differentiation medium with 5 ng/mL TNF-α and approximately 1.8 × 10^8^ CTXD14SkM-EVs. After 3 days of treatment, cells were evaluated for myosin heavy chain (MHC) expression.

### 2.6. Immunostaining of Cells

C2C12 were fixed with 4% paraformaldehyde (PFA) for 10 min at room temperature, followed by permeabilization with 0.1% Triton X-100 in PBS. Cells were then blocked in 10% FBS in PBS for 1 h at room temperature. The primary antibody, MF20 (Developmental Studies Hybridoma Bank, DSHB, Iowa, IA, USA), was diluted in a blocking buffer and applied overnight at 4 °C. The following day, cells were washed three times with PBS to remove any excess primary antibodies. A secondary antibody, donkey anti-mouse (Abcam, Waltham, MA, USA, ab150108) diluted in PBS, was then applied for 1 h at room temperature. After incubation, cells were washed three additional times with PBS, and DAPI was applied for nuclear counterstaining. Fluorescence images were captured using a fluorescence microscope.

### 2.7. Histology

TA muscle tissues were embedded in the OCT compound and frozen. Cryosections (10 µm) were prepared, mounted on glass slides, and air-dried. Sections were fixed in 4% PFA for 10 min, rinsed in PBS, and stained with hematoxylin (Harris, Sigma-Aldrich, St. Louis, MO, USA, HHS32) for 1 min. After rinsing, sections were stained with eosin (Sigma-Aldrich, St. Louis, MO, USA, HT110316) for 30 s, followed by a brief wash in distilled water. Slides were then dehydrated through graded ethanol concentrations, cleared with xylene, and coverslipped with a permanent mounting medium. Images were captured using a light microscope.

### 2.8. Immunohistochemistry of Skeletal Muscle

TA muscle tissues were embedded in OCT, frozen, and cryosectioned at 10 µm thickness. Sections were mounted on glass slides, air-dried, and fixed with 4% PFA for 10 min, followed by PBS rinses. Tissues were permeabilized with 0.1% Triton X-100 in PBS for 10 min, then blocked in 10% fetal bovine serum (FBS) in PBS for 1 h at room temperature. Primary antibody against Laminin (Sigma-Aldrich, St. Louis, MO, USA, L9393, 1:1000) was applied in the blocking buffer and incubated overnight at 4 °C. The next day, sections were washed in PBS and incubated with fluorophore-conjugated secondary antibodies in PBS for 1 h at room temperature in the dark. After the final PBS washes, sections were mounted with an antifade mounting medium containing DAPI for nuclear counterstaining. Images were captured using a fluorescence microscope. The CSA of muscles was measured by ImageJ software. For M1/2 macrophage staining, the longitudinal section of GAS muscle, 20 µm thick, was collected and fixed in 4% PFA for 10 min. Tissues were then permeabilized with 0.1% Triton X-100 in PBS for 10 min, followed by blocking in the solution of 10% FBS and 0.1% Triton X-100 in PBS for 1 h at room temperature. Primary antibodies anti-CD11b (eBioscience, San Diego, CA, USA, 14-0112-81, 1:100), iNOS (Bio-Techne, Minneapolis, MN, USA, NB300-605SS, 1:200), and anti-mannose receptor (Abcam, Waltham, MA, USA, ab64693, 1:1000), were prepared in the blocking buffer and applied to the tissue sections, which were incubated overnight at 4 °C. The following day, sections were washed with PBS and incubated with fluorophore-conjugated secondary antibodies: Alex Fluor 488 anti-Rat (Thermo Fisher Scientific, Waltham, MA, USA, 53-4031-80, 1:1000) and Alex Fluor 594 anti-rabbit (Abcam, Waltham, MA, USA, ab150080, 1:1000) for 1 h at room temperature in the dark. Finally, images were captured using a fluorescence microscope.

### 2.9. Western Blotting

Collected TA muscle samples were lysed in RIPA buffer (Sigma-Aldrich, St. Louis, MO, USA, 20-188) supplemented with phosphatase and protease inhibitors. Proteins were separated via SDS-PAGE gel, transferred onto PVDF membranes, blocked with 5% FBS in PBST (PBS with 0.1% Tween 20) for 1 h, and then incubated overnight at 4 °C with primary antibodies: anti-myogenin (Thermo Fisher Scientific, Waltham, MA, USA, PA5-87235, 1:1000), anti-NF-κB p65 (Cell Signaling Technology, Danvers, MA, USA, 8242, 1:1000), anti-Phospho-NF-κB p65 (Cell Signaling Technology, Danvers, MA, USA, 3033, 1:1000), anti-beta-actin (Cell Signaling Technology, Danvers, MA, USA, 4970S, 1:1000), anti-Vinculin (Cell Signaling Technology, Danvers, MA, USA, 2148S, 1:1000), and anti-GAPDH (Cell Signaling Technology, Danvers, MA, USA, 97166T, 1:1000). Membranes were then incubated with HRP-conjugated anti-rabbit IgG (H+L) secondary antibody (Promega, Madison, WI, USA, W4011, 1:10,000) or HRP-conjugated anti-mouse IgG (Cell Signaling Technology, Danvers, MA, USA, 7076S, 1:10,000) for 1 h at room temperature. Protein bands were revealed using a WesternBright enhanced chemiluminescent system (ECL, Thermo Fisher Scientific, Waltham, MA, USA, NC0930892). Images were acquired by using the ChemiDoc™ MP imaging system (Bio-Rad, Hercules, CA, USA) and analyzed by ImageJ software version 1.53K.

### 2.10. RNA Extraction and Real-Time PCR

Total RNA was extracted from TA muscle using the TRIzol–chloroform method. Tissue samples were homogenized in 500 µL TRIzol until fully lysed, followed by a 5 min incubation at room temperature. After adding 125 µL chloroform and vigorous shaking, the samples were incubated for 5 min and centrifuged at 10,000 rpm for 5 min to separate phases. The RNA-containing aqueous layer (~250 µL) was carefully collected and precipitated with 275 µL isopropanol, incubated for 5 min, and centrifuged at 14,000 rpm for 20 min at 20 °C. The RNA pellet was washed twice with 75% ethanol, centrifuged at 9500 rpm, and air-dried before being resuspended in 30 µL DNase/RNase-free water. Subsequently, 500 ng of total RNA was reverse transcribed into complementary DNA (cDNA) using the GoScript Reverse Transcriptase kit (Promega, Madison, WI, USA, A5003). Quantitative real time-PCR (qRT-PCR) was performed to measure messenger RNA (mRNA) expression of IL-10 (forward primer: TGGACAACATACTGCTAACCGAC; reverse primer: CCTGGGGCATCACTTCTACC), Chil3 (forward primer: TTTCTCCAGTGTAGCCATCCTT; reverse primer: TCTGGGTACAAGATCCCTGAA), and Arg1 (forward primer: CTCCAAGCCAAAGTCCTTAGA; reverse primer: AGGAGCTGTCATTAGGGACATC), with GAPDH (forward primer: CACCATCTTCCAGGAGCGAG; reverse primer: CCTTCTCCATGGTGGTGAAGAC) as the endogenous control. qRT-PCR was conducted on a 7300 Real-Time PCR system (Thermo Fisher Scientific, Waltham, MA, USA) using the Tribo 2x SYBR qPCR Super Mix Lox Rox (TBS4001LR-20) (Tribioscience, Sunnyvale, CA, USA).

### 2.11. Statistical Analysis

Data were analyzed using GraphPad Prism version 10.0 (GraphPad Software). A two-tailed, unpaired Student’s Paired *t*-test was used for comparisons between the two treatment groups. For comparisons among more than two groups with a single variable, one-way ANOVA followed by Tukey’s post hoc test was applied for multiple comparisons. All data are presented as mean ± standard error of the mean (SEM). Statistical significance was set at * *p* ≤ 0.05, ** *p* ≤ 0.005, *** *p* < 0.0001, **** *p* ≤ 0.0001.

## 3. Results

### 3.1. Isolation and Characterization of CTXD14SkM-EVs

**Figure 1:**
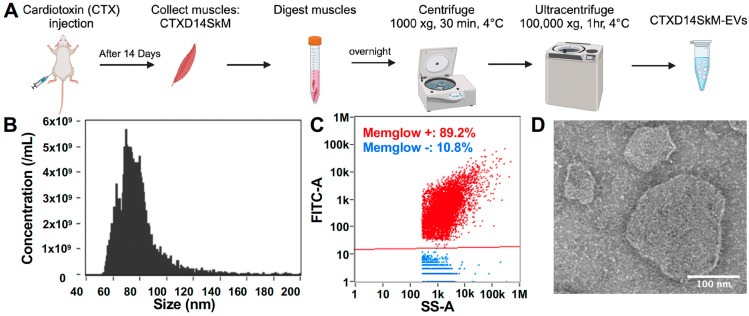
Isolation and characterization of CTXD14SkM-EVs: (**A**) Schematic overview of the isolation process for CTXD14SkM-EVs. (**B**) Size distribution of EVs. (**C**) MemGlow staining of EVs: red dots represent the MemGlow-positive population, and blue dots represent the MemGlow-negative population. Side scatter (SS) and FITC intensity of the EVs were detected using NanoFCM. (**D**) Contrast image of CTXD14SkM-EVs sample. Scale bar: 100 µm.

To isolate and enrich CTXD14SkM-EVs, skeletal muscle from 3-month-old wild-type mice was dissected 14 days after acute injury induced by intramuscular injection of CTX. The muscles underwent enzymatic dissociation, filtration, and sequential ultracentrifugation (Figure 1A). Nanoflow cytometry was used to assess the concentration and size distribution of these isolated EVs, confirming a size range of 50–200 nm, consistent with reported extracellular vesicles, particularly exosomes (Figure 1B). These isolated EVs were then treated with MemGlow dye to label the lipid bilayer, and nanoflow cytometry-based analysis revealed that around 90% of the particles are fluorescent and lipid-enclosed structures, indicating the high purity of these EV samples (Figure 1C). Compared to EVs derived from wild-type mouse skeletal muscle, these derived from acutely injured skeletal muscle showed no significant difference in size distribution (Figure S1). In addition, the morphological integrity and size of CTXD14SkM-EVs were further identified by TEM (Figure 1D).

### 3.2. CTXD14SkM-EVs Promote Myoblast Differentiation in a Cellular Muscle Atrophy Model Induced by Pro-Inflammatory Cytokine TNF-α

**Figure 2:**
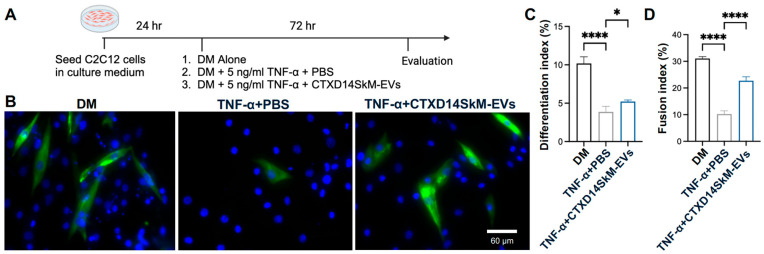
CTXD14SkM-EVs promote myoblast differentiation in a cellular muscle atrophy model induced by pro-inflammatory cytokine TNF-α: (**A**) Schematic overview of C2C12 differentiation. (**B**) Immunostaining of MHC (green) and DAPI (blue) in three groups: differentiation medium only, differentiation medium with 5 ng/mL TNF-α and PBS, differentiation medium with 5 ng/mL TNF-α and CTXD14SkM-EVs. Scale bar: 60 µm. (**C**) Differentiation index calculated as (number of MHC+ cells/total number of nuclei). (**D**) The Fusion index is calculated as (number of nuclei in MHC+ cells with ≥2 nuclei/total number of nuclei). The experiment was repeated, and data were reported as means ± SEM. * *p* ≤ 0.05, **** *p* < 0.0001 by one-way ANOVA.

To investigate the therapeutic potential of CTXD14SkM-EVs in mitigating muscle atrophy, we employed a well-established myotube atrophy model using C2C12 myoblasts. This model was induced by TNF-α, a pro-inflammatory cytokine elevated in ALS and other muscle-wasting disorders. TNF-α is known to impair myogenic differentiation and activate catabolic pathways, including the ubiquitin-proteasome system and NF-κB signaling, leading to the downregulation of essential myogenic markers such as myoblast determination protein 1 (MyoD) and myogenin, while promoting the degradation of muscle structural proteins [17,18,19]. Thus, TNF-α-induced muscle atrophy models are widely used to study muscle degeneration and evaluate therapeutic interventions. In this study, TNF-α was administered during C2C12 myoblast differentiation to inhibit myogenic progression and induce an atrophic state. To assess the protective effects of CTXD14SkM-EVs, these EVs were added concurrently with TNF-α. After 72 h of treatment, myotubes were immunostained for myosin heavy chain (MHC), a hallmark of mature muscle fibers (Figure 2A,B). Our results showed that TNF-α treatment markedly reduced both myoblast differentiation and fusion indices, consistent with its known atrophic effects. Strikingly, CTXD14SkM-EVs treatment significantly rescued both indices under TNF-α-induced condition, indicating a protective effect on myogenic differentiation and myotube formation (Figure 2C,D).

### 3.3. CTXD14SkM-EVs Alleviate ALS-Related Muscle Atrophy in SOD1^G93A^ Mice

**Figure 3:**
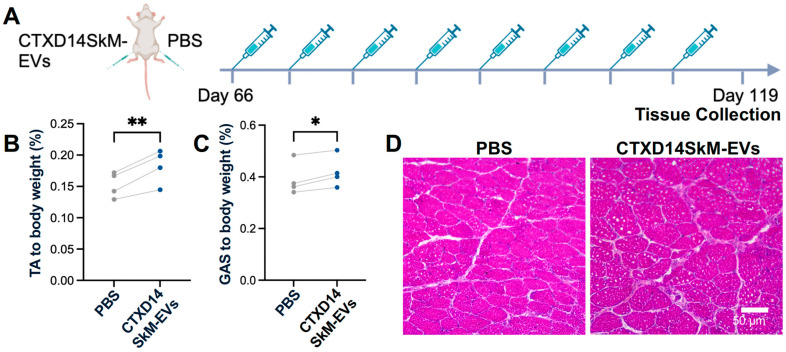
CTXD14SkM-EVs alleviate muscle atrophy in SOD1^G93A^ mice: (**A**) Schematic overview of CTXD14SkM-EVs and PBS treatment in SOD1^G93A^ mice. (**B**) TA muscle weight to body weight ratio (*n* = 4). Paired Student’s *t*-test. ** *p* ≤ 0.005. (**C**) GAS muscle weight to body weight ratio (*n* = 4). Paired Student’s *t*-test. * *p* ≤ 0.05. (**D**) H&E staining of TA muscle in two groups: SOD1^G93A^ muscle treated with PBS and SOD1^G93A^ muscle treated with CTXD14SkM-EVs. Scale bar: 50 µm.

To assess the therapeutic effects of CTXD14SkM-EVs on mitigating inflammation-driven muscle atrophy in vivo, we utilized a well-established ALS mouse model expressing the human SOD1 protein with the pathogenic missense mutation G93A (SOD1^G93A^) [20]. The SOD1^G93A^ mice recapitulate ALS-related muscle atrophy, accompanied by inflammation and impaired muscle regeneration. CTXD14SkM-EVs were intramuscularly injected into the TA and GAS muscles of one limb in SOD1^G93A^ mice, while the contralateral limb received PBS as a control. The treatment began at the pre-symptomatic stage (postnatal day 66, P66) and continued weekly until muscle tissues were collected at the late symptomatic stage (P119) for analysis (Figure 3A). First, the muscle weight-to-body weight ratios for the TA and GAS muscles were evaluated. Notably, both the TA and GAS muscles treated with CTXD14SkM-EVs exhibited significantly increased muscle weights compared to their PBS-treated counterparts (Figure 3B,C). Hematoxylin and eosin (H&E) staining of TA muscle fibers revealed that the CTXD14SkM-EVs treated group showed increased muscle fiber size and a higher abundance of centrally located myonuclei compared to PBS-treated muscles in SOD1^G93A^ mice (Figure 3D).

**Figure 4:**
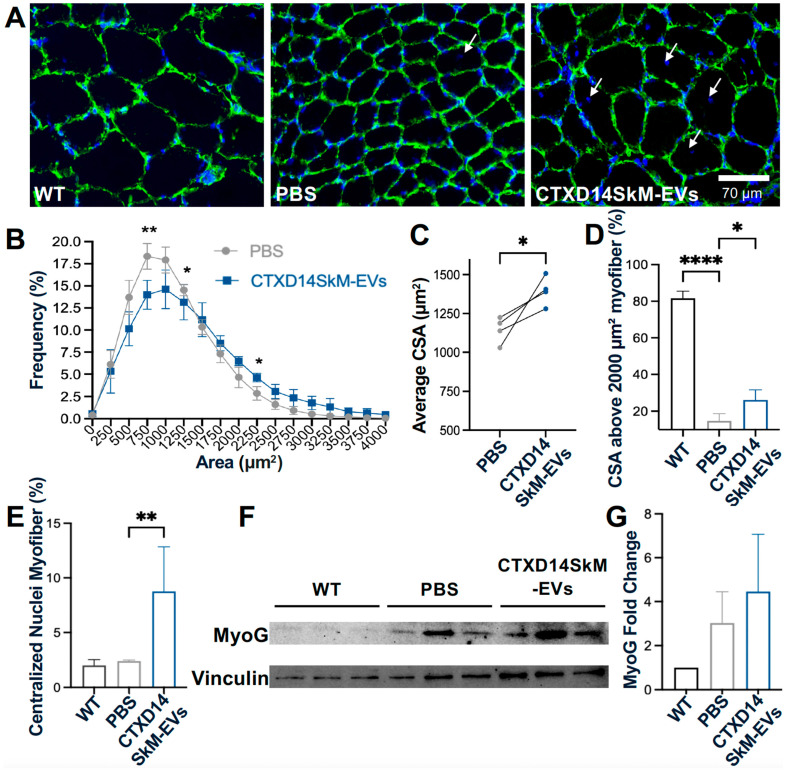
CTXD14SkM-EVs promote muscle regeneration in SOD1^G93A^ mice: (**A**) Immunostaining of Laminin (green) and DAPI (blue) in the three groups: WT muscle (*n* = 3), PBS (*n* = 4), CTXD14SkM-EVs (*n* = 4). Scale bar: 70 µm. White arrows indicate the centralized nuclei. CSA was measured by ImageJ. (**B**) Myofiber size distribution in two groups: PBS (gray, *n* = 4) and CTXD14SkM-EVs (blue, *n* = 4). (**C**) Comparison of average CSA between PBS and CTXD14SkM-EVs treated groups, analyzed by paired Student’s *t*-test. (**D**) Comparison of the percentage of CSA above 2000 µm^2^ among the three groups, analyzed by one-way ANOVA. (**E**) Percentage of myofibers with central nuclei to total myofibers, analyzed by one-way ANOVA. (**F**) Western blot analysis of myogenin and Vinculin protein levels in WT (*n* = 3), PBS (*n* = 3), and CTXD14SkM-EVs (*n* = 3) groups. (**G**) Myogenin and Vinculin protein levels among the three groups were quantified using ImageJ. The protein fold change was evaluated by normalizing myogenin levels to the housekeeping protein Vinculin. The results were analyzed by one-way ANOVA. * *p* ≤ 0.05, ** *p* ≤ 0.005, **** *p* < 0.0001.

To further investigate CSA distribution, immunostaining for Laminin, a myofiber basement member marker, was performed (Figure 4A). TA muscles from the CTXD14SkM-EV-treated group exhibited a reduced proportion of small CSA myofibers and an increased proportion of larger CSA myofibers compared to the PBS-treated control group (Figure 4B). The average CSA was significantly greater in the CTXD14SkM-EVs-treated group, showing about a 20% increase compared to the PBS-treated group (Figure 4C,D), indicating that CTXD14SkM-EVs may mitigate muscle atrophy in SOD1^G93A^ mice. Furthermore, the percentage of myofibers with centralized nuclei, an indicator of active regeneration, was significantly higher in the CTXD14SkM-EVs-treated group, showing an increase of about 6% compared to the PBS-treated group, suggesting that CTXD14SkM-EVs may promote muscle stem cell-mediated regeneration (Figure 4E). Additionally, the protein level of myogenin, a myogenic marker linked to the differentiation commitment of muscle stem cells, showed an increasing trend in the EV-treated TA muscle group, supporting the regenerative potential of CTXD14SkM-EVs (Figure 4F,G).

### 3.4. CTXD14SkM-EVs Enhance M2 Macrophage Polarization in ALS-Affected Skeletal Muscle

**Figure 5:**
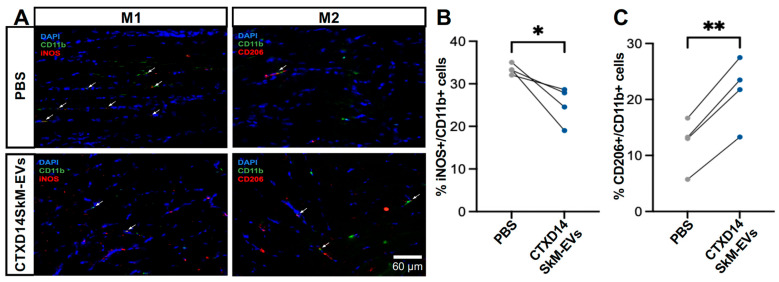
CTXD14SkM-EVs enhance M2 macrophage polarization in ALS-affected skeletal muscle: (**A**) Immunostaining of CD11b (green), iNOS (red), CD206 (red), and DAPI (blue) in GAS muscle sections from PBS (*n* = 4) and CTXD14SkM-EVs (*n* = 4) groups. Scale bar: 60 µm (**B**) Comparison of the percentage of M1 macrophage (iNOS+/CD11b+) between the PBS and CTXD14SkM-EVs groups, analyzed by paired Student’s *t*-test. (**C**) Comparison of the percentage of M2 macrophage (CD206+/CD11b+) between the PBS and CTXD14SkM-EVs groups, analyzed by paired Student’s *t*-test. * *p* ≤ 0.05, ** *p* ≤ 0.005.

Given the anti-inflammatory microenvironment predominantly driven by M2 macrophages in the skeletal muscles 14 days post-acute injury (CTXD14SkM), we hypothesized that EVs derived from CTXD14SkM would inherit the capacity to promote macrophage polarization towards an anti-inflammatory and pro-regenerative state [12,14]. To test this hypothesis, we performed immunostaining for M1 and M2 macrophage markers in the GAS muscles of SOD1^G93A^ mice treated with either CTXD14SkM-EV or PBS (Figure 5A). The results showed that CTXD14SkM-EV treatment significantly increased the proportion of M2 macrophages (CD206+/CD11b+) by approximately 9.6% while reducing the presence of M1 macrophages (iNOS+/CD11b+) by about 8.4% compared to PBS treatment (Figure 5B,C). These findings indicate that CTXD14SkM-EVs effectively promote the polarization of macrophages toward a pro-regenerative M2 phenotype.

### 3.5. CTXD14SkM-EVs Suppress NF-κB Pathway Activation in the Skeletal Muscle of ALS Mice

**Figure 6:**
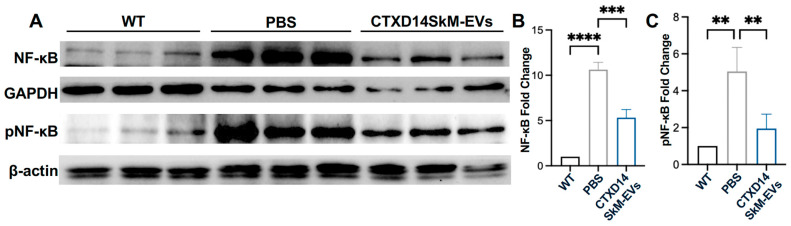
CTXD14SkM-EVs suppress NF-κB pathway activation in the skeletal muscle of SOD1^G93A^ Mice: (**A**) Western blot analysis of NF-κB, pNF-κB, GAPDH, and β-actin proteins for three groups: WT (*n* = 3), PBS (*n* = 3), and CTXD14SkM-EVs (*n* = 3). (**B**) Quantification of NF-κB protein levels among the three groups using Image J, statistical analysis by one-way ANOVA. (**C**) Quantification of pNF-κB protein levels among the three groups using Image J, statistical analysis by one-way ANOVA. ** *p* ≤ 0.005, *** *p* < 0.0001, **** *p* < 0.0001.

To assess the impact of CTXD14SkM-EVs treatment on NF-κB signaling in the TA muscle of SOD1^G93A^ mice, we performed Western blot analysis to quantify the expression of total NF-κB, the active phosphorylated format of NF-κB (pNF-κB), and the loading controls GAPDH and β-actin across three experimental groups: wild-type (WT) mice, PBS-treated ALS mice, and CTXD14SkM-EV-treated ALS mice. As expected, both NF-κB and pNF-κB protein levels were significantly elevated in the TA muscle of the PBS-treated ALS mice compared to the WT group, indicating a heightened inflammatory response in ALS-affected skeletal muscles. Notably, CTXD14SkM-EV treatment significantly reduced NF-κB and pNF-κB protein levels in ALS-affected muscle by approximately 50%, bringing them closer to baseline levels observed in WT mice (Figure 6A–C). Meanwhile, the CTXD14SkM-EV-treated group exhibited an increased expression of key markers associated with M2 macrophage polarization and anti-inflammatory responses, including Interleukin-10 (IL-10), Chitinase-Like Protein 3 (Chil3), and Arginase 1 (Arg1) (Figure S2). These findings suggest that CTXD14SkM-EV treatment effectively suppresses NF-κB signaling in the skeletal muscles of SOD1^G93A^ mice, supporting its potential to mitigate inflammation and muscle atrophy in ALS.

## 4. Discussion

This study explores the therapeutic potential of EVs derived from regenerating skeletal muscle for treating ALS-afflicted muscle atrophy. Most current pharmacological treatments for ALS focus on targeting neuronal deficits, showing limited clinical promise. Given the importance of muscle atrophy in ALS progression and pathogenesis, approaches targeting muscle wasting are of high therapeutic interest. Multiple pathogenic mechanisms are known to be involved in ALS-related motor defects and muscle atrophy, including chronic inflammation, impaired regeneration, proteostasis dysregulation, and mitochondrial and metabolic defects. Current therapeutic strategies targeting ALS-afflicted muscle defects often focus on one or a few misregulated pathways. Our findings demonstrated that regenerating skeletal muscle-derived EVs provide a multifaceted strategy to counter muscle atrophy in an ALS mouse model (SOD1^G93A^) by promoting muscle regeneration, shifting macrophage polarization towards an anti-inflammatory phenotype, and downregulating the pro-inflammatory NF-κB signaling pathway essential for protein homeostasis. These EVs (CTXD14SkM-EVs) are derived from cells within the regenerating skeletal muscle 14 days post-acute injury, the peak phase of regeneration facilitated by the collective activities of residing cells, such as macrophages predominantly in an anti-inflammatory M2 state, activated muscle stem cells, and newly formed muscle fibers. EVs are known to inherit molecular cargos from their sourced cells and mediate regulatory functions in recipient cells. Recently, EVs have been proven to be key mediators of the therapeutic effects of stem cells instead of stem cell engrafting and differentiation. As expected, the CTXD14SkM-EVs may carry and transfer functional molecules from cells within the regenerating skeletal muscle to recipient cells in ALS-affected skeletal muscle, facilitating an anti-inflammatory and pro-regenerative environment for muscle repair.

In ALS-like cellular models of muscle wasting, the CTXD14SkM-EVs mitigated the detrimental effects induced by the pro-inflammatory cytokine TNF-α on myoblasts, as evidenced by improved differentiation and fusion indices. Moreover, in vivo, intramuscular administration of CTXD14SkM-EVs alleviated ALS-related muscle atrophy in SOD1^G93A^ mice, as indicated by increased muscle mass and muscle fiber size. Meanwhile, a higher percentage of myofibers with centralized nuclei was observed post EV treatment, suggesting active muscle regeneration. These results suggest that CTXD14SkM-EVs may counteract inflammation-induced muscle wasting by enhancing myoblast function and promoting muscle regeneration.

The anti-inflammatory properties of CTXD14SkM-EVs were further supported by their ability to modulate macrophage polarization and regulate NF-κB signaling. In ALS-affected skeletal muscle, chronic inflammation involves a disrupted transition of macrophages from the pro-inflammatory M1 phenotype, which clears debris and damaged tissue, to the anti-inflammatory M2 phenotype, which promotes muscle regeneration and tissue repair. This prolonged pro-inflammatory state may not only impair muscle stem cell function for regeneration but also exacerbate muscle protein breakdown, leading to muscle atrophy [21,22]. CTXD14SkM-EVs effectively promoted a phenotypic shift in macrophages from the M1 to M2 phenotype in ALS-affected skeletal muscle. This shift may alleviate the inflammatory burden and create a more favorable microenvironment for muscle regeneration and repair. As such, CTXD14SkM-EVs hold significant potential as modulators of immune responses in ALS therapy. In addition to modulating macrophage populations, CTXD14SkM-EVs also significantly suppressed the excessive activation of the NF-κB pathway in ALS skeletal muscles. Specifically, they downregulated the expression of both NF-κB and its phosphorylated form (pNF-κB) in the skeletal muscles of SOD1^G93A^ mice. Aberrant activation of NF-κB signaling in ALS-afflicted skeletal muscles is a key driver of chronic inflammation, suppression of myogenic regulatory factors, and upregulation of proteolytic enzymes, collectively contributing to muscle degeneration and wasting [6,8,23,24,25,26]. Thus, targeting NF-κB signaling represents a promising therapeutic strategy to attenuate inflammation and mitigate muscle atrophy in ALS. By downregulating NF-κB signaling, CTXD14SkM-EVs may interrupt these pathogenic cascades, thereby reducing chronic inflammation, enhancing myogenic differentiation, and decreasing protein degradation. Together, these therapeutic effects contribute to muscle repair and functional recovery in ALS.

This study demonstrates that CTXD14SkM-EVs possess therapeutic potential to mitigate ALS-related muscle atrophy, providing valuable insights into leveraging EVs derived from regenerating skeletal tissue to address multiple pathogenic processes underlying muscle degeneration. These findings lay a strong foundation for developing EV-based therapies targeting muscle atrophy in ALS, which could complement therapeutic strategies focusing on neuroprotection. Future research should prioritize identifying the molecular cargo within CTXD14SkM-EVs responsible for these therapeutic effects, as well as optimizing delivery and dosing strategies to enhance their efficacy in treating ALS and other muscle-wasting diseases.

## Data Availability

The data presented in this study are available on request from the corresponding author since they are related to ongoing project study.

## Supplementary Materials

The following supporting information can be downloaded at: https://www.mdpi.com/article/10.3390/cells14060464/s1, Figure S1: Characterization of SkM-EVs derived from wild-type mouse (3-month-old); Figure S2: mRNA expression levels of anti-inflammatory factors IL-10, Chil3 and Arg1 were compared between control (PBS) and EV (CTXCD14SkM-EVs) treated skeletal muscles of SOD1G93A Mice.
