# Supplementary material for: Extracellular Vesicles from Regenerating Skeletal Muscle Mitigate Muscle Atrophy in an Amyotrophic Lateral Sclerosis Mouse Model
Source: Cells. 2025 Mar 20;14(6):464. doi: 10.3390/cells14060464 (PMC11941016; doi:10.3390/cells14060464)
Supplement: Supplementary file 1 [file cells-14-00464-s001.zip › cells-3490060-supplementary.pdf]

Supporting Information

Extracellular Vesicles from Regenerating Skeletal Muscle Mitigate Muscle Atrophy in an Amyotrophic Lateral Sclerosis Mouse Model

Jinghui Gao[1], Aria Sikal[1], Rachel Hankin[1], Yaochao Zheng[1], Elijah Sterling[1], Kenny Chan[2] and Yao Yao[1 *]

[1]Regenerative Bioscience Center, Department of Animal and Dairy Science, College of Agricultural and Environmental Science, University of Georgia, Athens, GA 30602, USA
[2]Department of Physiology and Pharmacology, College of Veterinary Medicine, University of Georgia, Athens, GA 30602, USA

*Correspondence: yao.yao@uga.edu

Supplementary Figures

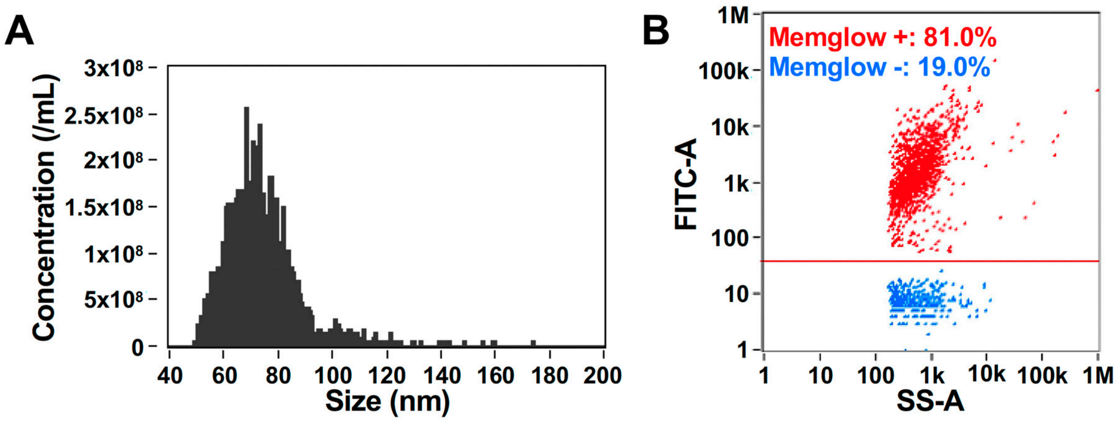

Figure S1. Characterization of SkM-EVs derived from wild-type mouse (3-month-old). Size distribution (A) and MemGlow staining of EVs (B) were analysed by NanoFCM. Red dots represent the MemGlow-positive population, blue dots represent the MemGlow-negative population. Side scatter (SS) and FITC intensity of the EVs were detected using NanoFCM.

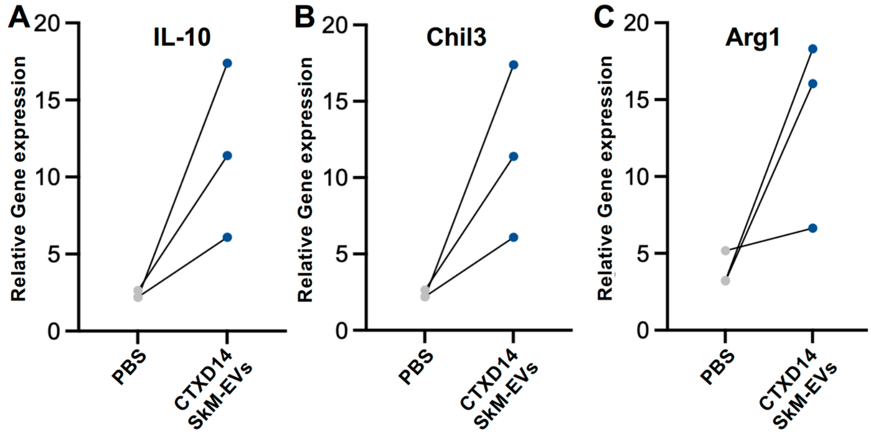

Figure S2. mRNA expression levels of anti-inflammatory factors IL-10, Chil3 and Arg1 were compared between control (PBS) and EV (CTXCD14SkM-EVs) treated skeletal muscles of SOD1^{G93A} Mice. (n = 3). Statistical analysis was performed using a paired t-test.
